# Biochemical markers and FokI and TaqI vitamin D receptor genes polymorphism in rheumatoid arthritis

**DOI:** 10.1186/s12920-023-01668-8

**Published:** 2023-10-19

**Authors:** Hosam M. Ahmad, Zaki M. Zaki, Asmaa S. Mohamed, Amr E. Ahmed

**Affiliations:** 1 Biotechnology and life sciences department, faculty of post graduate studies for advanced sciences, Beni-Suef university, Beni-Suef, Egypt; 2 Internal Medicine and Biomedical Chemistry Departments, Ministry of Health and population, Minia, Egypt; 3 Clinical Pathology department, Faculty of Medicine, Minia University, Minia, Egypt; 4 Clinical Pharmacy and pharmacy practice department, Faculty of Pharmacy, Port said University, Port said, Egypt

**Keywords:** Rheumatoid arthritis, Gene polymorphism, CRP, ESR, Rheumatoid factor

## Abstract

**Background:**

Previous studies have reported the role of genes in different metabolic processes in the human body, and any variation in gene polymorphisms could lead to disturbances in these processes and different diseases.

**Objective:**

This study aimed to compare vitamin D receptor (VDR) FokI and TaqI genotypes in terms of parathyroid hormone (PTH) and some biomarkers of inflammation and susceptibility to rheumatoid arthritis (RA) disease.

**Methods:**

This study included 100 patients with rheumatoid arthritis (RA). Genotyping was performed by polymerase chain reaction (PCR) and examined by specific restriction enzymes using restriction fragment length polymorphism (RFLP). Serum intact PTH, C-reactive protein (CRP), erythrocyte sedimentation rate (ESR), rheumatoid factor (RF), and anti-cyclic citrullinated peptide antibodies (ACCPs) levels were measured.

**Results:**

An increased PTH level (> 65 pg/ml) was found in 8% of patients. No significant differences among FokI and TaqI vitamin D receptor genes polymorphism regarding positive and negative RF or ACCPs were found. A significant difference was found among FokI (p = 0.009) and none in TaqI genotypes regarding intact parathyroid hormone level categories. No significant correlation was found between the serum intact PTH level and ESR or CRP levels (P = 0.13 and 0.28, respectively). The parathyroid hormone level was not a good predictor for RF or ACCPs (P = 0.5 and 0.06, respectively).

**Conclusion:**

The FokI gene may play a role in controlling PTH levels in patients with RA. There was no significant correlation found between the serum intact PTH level and RA severity according to ESR and CRP inflammatory biomarkers. There are no differences between VDR genes FokI and TaqI polymorphism in terms of RA susceptibility (for RF and ACCPs).

## Introduction

**Fig. 1:**
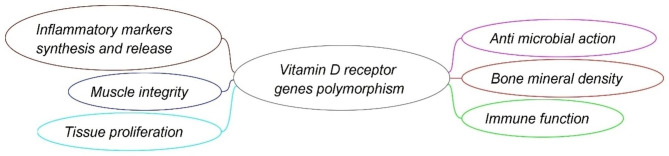
Some bio-functions of vitamin D receptor genes

Sensitivity to vitamin D supplementation is influenced by genetic variations in the vitamin D receptor (VDR) genes and is controlled by both genetic and environmental factors [1]. The VDR genes provide instructions for making a protein called vitamin D receptor (VDR), which allows the body to respond to vitamin D. Vitamin D receptor genes may be responsible for some important biological functions in the human body [2] (Fig. 1). Diet, sun exposure, pathogens, and pollution are the main environmental factors linked to VDR regulation [3–6].

Rheumatoid arthritis (RA) is a chronic inflammatory disorder that affects the joints and occurs when the immune system unintentionally damages tissues in the body [7]. Rheumatoid arthritis is linked to comorbid diseases, altered skeletal bone metabolism, and joint inflammation. Maintaining function and avoiding disability are possible with early diagnosis and effective treatment that reduces the progression of the disease [8].

Research is being conducted on the use of biochemical markers for joint cartilage and bone degeneration that might enable early detection and assessment of joint injuries [8]. The first autoantibody associated with RA is rheumatoid factor (RF) [9]. When rheumatoid factor and IgG combine, immune complexes are formed, causing the disease [10]. Rheumatoid factor can be any isotype of immunoglobulins, for example, IgA, IgG, IgM, IgE, or IgD, while being most frequently seen as IgM [11–13]. High levels of rheumatoid factor are found in rheumatoid arthritis (80%) and Sjögren’s syndrome (70%) [14].

Several polymorphisms of the human VDR gene FokI have resulted in VDR proteins with diverse structures, such as extended f-VDR or smaller f-VDR. Immune cell function is influenced by VDR FokI polymorphism, and perhaps FokI polymorphism plays a role in immune-related diseases [15]. Many studies have evaluated the relationship between the VDR FokI polymorphism and the inherited propensity for bone disorders [16], risk of malignancy [17], and immunological diseases [18–20].

Studies on different VDR gene polymorphisms that were conducted on the TaqI polymorphism revealed an association with the risk of breast cancer, [21] as well as with the onset of arthritis [22]. The studied TaqI polymorphism did not show any association with type 1 diabetes mellitus at allelic or genotypic levels, [23] nor with developing a risk for urolithiasis [24].

Anti-cyclic citrullinated peptide antibodies (ACCPs) have recently been used to classify rheumatoid arthritis (RA). This antibody is more specific than rheumatoid factor (RF) for the diagnosis of RA [25]. It holds promise for earlier and more accurate diagnosis of the disease, improved prognostic information, and has been implicated in RA pathogenesis [26]. Testing of ACCPs showed sensitivity ranging from 39 to 50% and specificity from 93 to 98% in patients who were eventually diagnosed with RA, compared to other non-RA patients [27–32]. The combination of RF and ACCPs predicted RA with good sensitivity and specificity [26].

C-reactive protein (CRP) and erythrocyte sedimentation rate (ESR) are two of the earliest tests performed in laboratories that remain in use [33, 34]. Both blood tests are used to detect inflammation in the body [35–37]. As a result of the acute phase reaction, CRP is formed in the liver. It responds to alterations in the inflammatory process and is immediately measurable. A quick peak in CRP levels occurs 48 h following the inflammatory stimulation. The CRP level drops quickly when the trigger for production is removed [35].

ESR is a sensitive indicator of inflammation. Inflammation causes a steady increase in ESR levels, and it may take weeks for these levels to drop to baseline [38]. ESR is affected by multiple variables, including physiological factors (e.g., older age, female gender, and pregnancy) [35, 38–40], and pathological factors (e.g., plasma immunoglobulin and fibrinogen concentrations). Therefore, in order to investigate an inflammatory clinical condition, CRP is the chosen test [41]. Due to the slower reaction rate and lack of specificity of ESR in comparison to CRP, monitoring ESR might produce false-negative or false-positive results [38].

Parathyroid glands secrete parathyroid hormone (PTH). It controls the level of calcium by affecting calcium metabolism through intestinal absorption, renal excretion, and bone mineralization [42]. The chief cells of the parathyroid glands are principally responsible for PTH secretion. Chromosome 11 contains the PTH gene. It is a polypeptide prohormone with 84 amino acids. The hormone calcitonin counteracts the effects of PTH [43].

The aim of this study is to compare the vitamin D receptor (VDR) FokI and TaqI genotypes in terms of parathyroid hormone (PTH) and selected biomarkers of inflammation and susceptibility in rheumatoid arthritis disease.

## Methods

### Study design

This descriptive study was conducted at the special rheumatology clinics at Minia Hospital, Minia Governorate, Egypt.

### Participants

One hundred outpatients previously diagnosed with rheumatoid arthritis who fulfilled the 2010 “American College of Rheumatology/European League against Rheumatism classification criteria for RA patients” [44] were selected randomly.

**Fig. 2:**
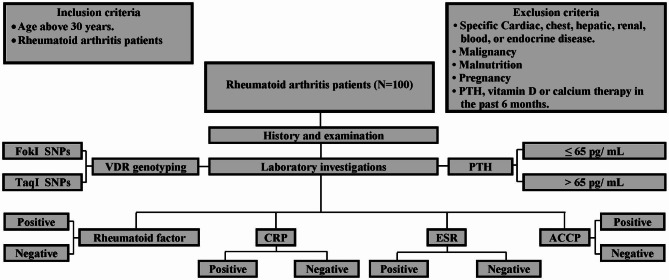
Flow diagram of the study

Age was over thirty years. All patients fulfilled the inclusion and exclusion criteria (Fig. 2).

### Procedures

The study sample was selected by consecutive sampling. All patients were subjected to a complete medical history, examination, and laboratory investigations. The main study steps are summarized in Fig. 2. The age of onset of RA in the study sample ranged from 29 to 44 years. The disease duration was between one and 16 years. The treatments differed among the study sample. Examples of treatments included Methotrexate, Leflunomide and Hydroxychloroquine.

### Outcomes and measures

CRP, ESR, RF, ACCPs, and PTH levels were measured. The normal range of intact parathyroid hormone is 10–65 pg/ml; for the study purpose, we categorized it into ≤ 65 pg/ml and > 65 pg/ml categories. Both RF and ACCPs levels were categorized as positive (+ ve) or negative (-ve). ESR was determined by the Westergren method. CRP and RF levels were assessed using a semiautomated method - latex agglutination test (spinreact – Spain). PTH level was assessed using Cobas e 411 analyzer (Roche, USA), ACCPs level was assessed using TOSOH AIA 360 automated immunoassay analyzer (Japan).

Relationships among the VDR FokI (rs2228570), TaqI (rs731236) genotypes, RF, ACCPs, and categorical PTH levels were compared.

### Genotyping

The FokI (rs2228570) and TaqI (rs731236) polymorphisms were analyzed using polymerase chain reaction (PCR) - restriction fragment length polymorphism (RFLP). Genomic DNA was extracted from peripheral white blood cells using the salting out procedure and amplified using the following forward and reverse primers:

FokI (F: 5’-AGCTGGCCCTGGCACTGACTCTGCTCT-3’ –.

R: 5’-ATGGAAACACCTTGCTTCTTCTCCCTC-3’)

TaqI (F: 5′-CAGAGCATGGACAGGGAGCAAG-3′ -.

R: 5′-CGGCAGCGGATGTACGTCTGCAG-3)

PCR amplification was performed using BIO RAD PCR kit (California, USA): 12.5 µl master mix, 1 µl of both forward and reverse primers, 0.5 µl of polymerase enzyme, 0.5 l templet DNA (salting out), and 7 µl of nuclease-free water.

PCR was performed using a (BIO RAD T100 Thermal Cycler - California, USA) with cycling condition of initial denaturation at 95˚C for 5 min, 40 cycles of denaturation at 95 ˚C for 45 s, annealing at 62.5˚C for 45 s, and extension at 72˚C for 45 s, followed by a final extension at 72˚C for 3 min. PCR products were separated on a 1.5% agarose gel with PCR marker (ladder) of 100 bp. The restriction endonucleases (Thermo Scientific FastDigest FokI restriction enzyme and Thermo Scientific FastDigest TaqI restriction enzyme - Lithuania) were used to digest the polymorphic sites of the VDR gene.

The enzyme-specific restriction reactions were performed at 37 °C for 5 min for the FokI, enzyme, and at 65ºC for 5 min for the TaqI enzyme. Digestion steps: The following components were mixed at room temperature: 0.5 l restriction enzyme, 1 l 10x buffer, 8 µl amplified PCR product, and 2 µl nuclease free water.

The sizes of the digested fragments were identified by 4% Bio Rad agarose gel electrophoresis, and the results were visualized under UV light and photographed. The size of the digested PCR products is as follows.

**Fig. 3:**
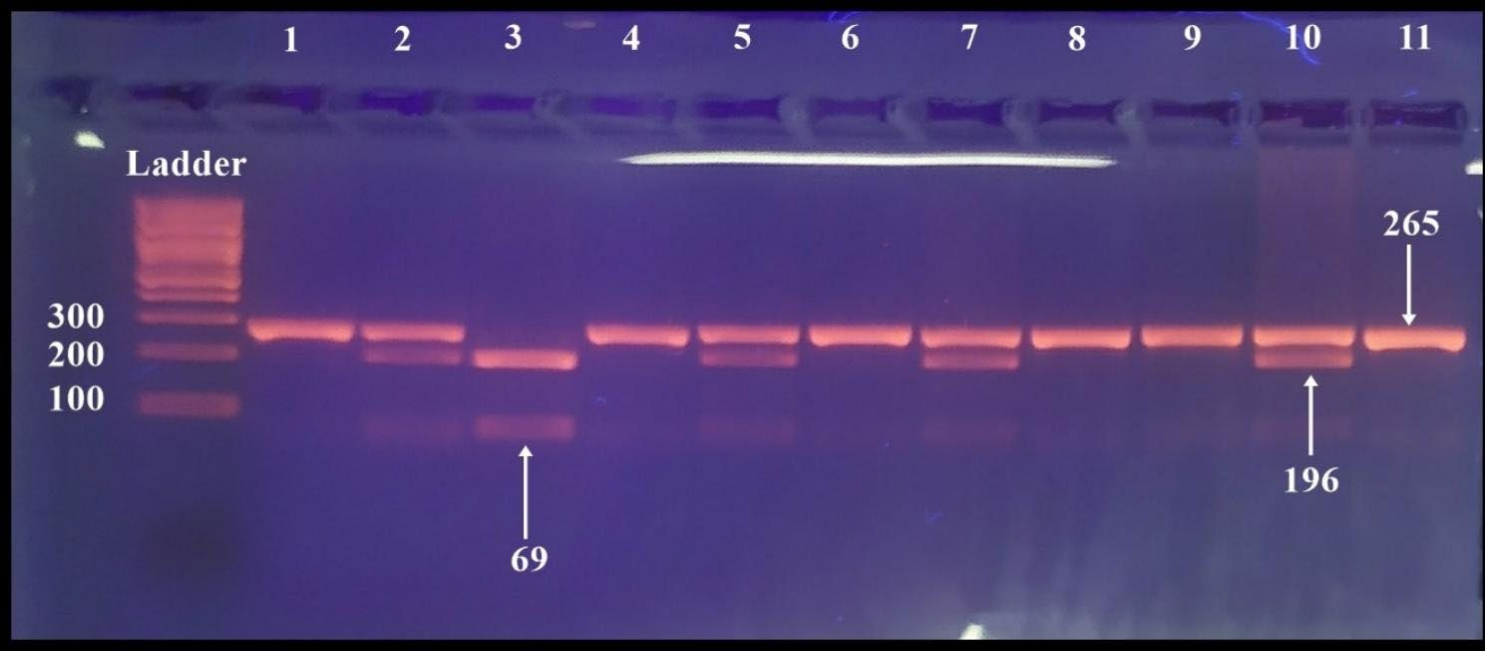
Gel electrophoresis of the PCR-RFLP technique of amplified FokI genotypes. Ladder of (100 bp). Lanes 1, 4, 6, 8, 9, and 11 represent homozygous FF genotype showing one fragment. Lane 3 represents the homozygous ff genotype showing two fragments. Lanes 2, 5, 7, and 10 represent heterozygous Ff genotype showing three fragments

For FokI: the undigested fragment (265 bp) specified the presence of the F allele, and the appearance of two fragments (196 bp and 69 bp) specified the presence of the f allele. The homozygous FF genotype led to one band at 265 bp, the homozygous ff genotype led to two bands at 196 bp and 69 bp, and the heterozygous Ff genotype led to three bands at 265, 196, and 69 bp (Figure 3).

**Fig. 4:**
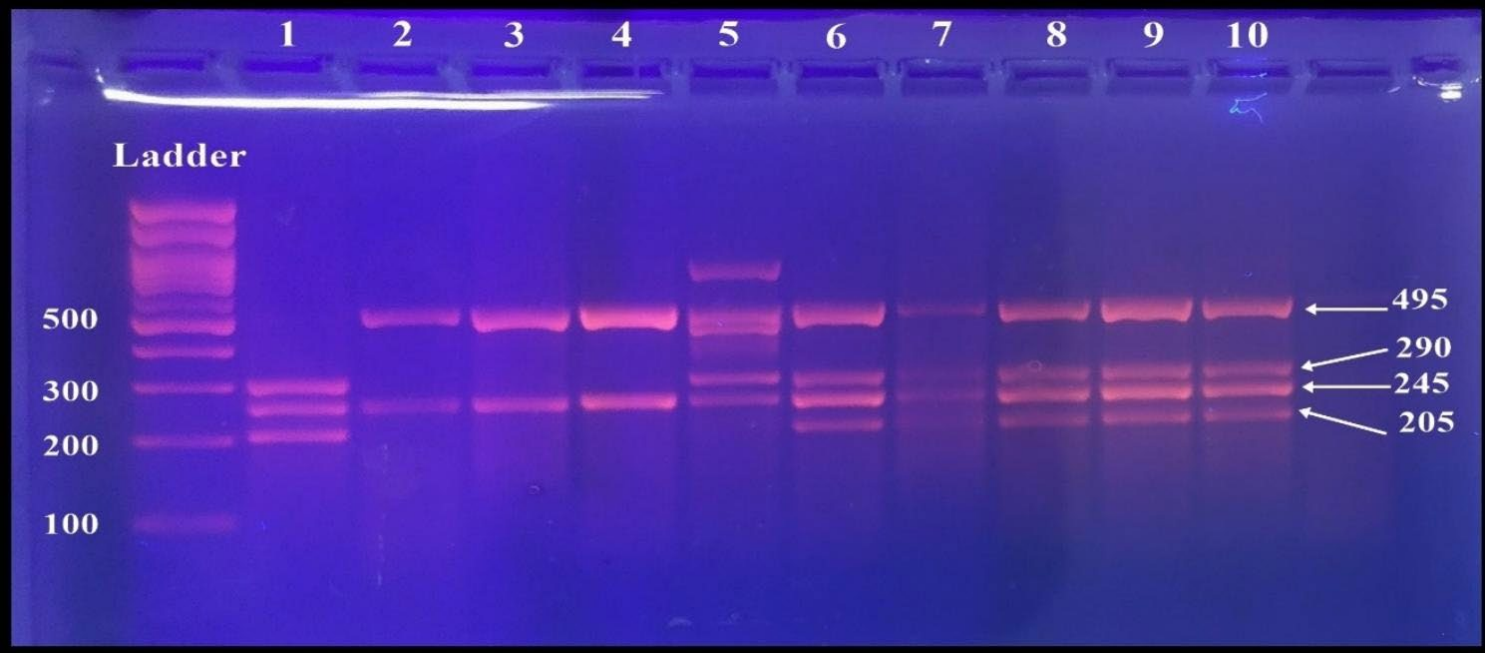
Gel electrophoresis of PCR-RFLP technique of amplified TaqI genotypes. Ladder of (100 bp). Lanes 2–4 represent homozygous TT genotype showing two bands. Lane 1 represents the homozygous tt genotype showing three bands. Lanes 5,6, and 8–10 represent heterozygous Tt genotype showing four bands

For TaqI: one fragment (245 bp) and an undigested one (495 bp) specified the presence of the T allele, and the appearance of three fragments (290 bp, 245 bp, and 205 bp) specified the presence of the t allele. The homozygous TT genotype led to two bands at 495 bp and 245 bp, the homozygous tt led to three bands at 290 bp, 245 bp, and 205 bp, and the heterozygous Tt led to four bands at 495 bp, 290 bp, 245 bp, and 205 bp (Figure 4).

### Statistical analysis

SPSS program version 26 statistical software was used to analyze the data. Results are presented as counts and percentages for categorical variables, or means and standard deviation (M ± SD) for continuous variables. The Kolmogorov–Smirnov test and Shapiro–Wilk test were used to test the normality of the data. Chi-Square and Fisher’s exact tests were used for comparing categorical data. In order to assess the significance and direction of the association between two variables, the Pearson correlation coefficient (r) was used. MedCalc version 20 statistical software was used to analyze the receiver operating characteristic (ROC) curve. P-value < 0.05 was set as the threshold of statistical significance.

## Results

This study included 100 rheumatoid arthritis patients. 18% were male, and 82% were female.

Table (1) shows that the M ± SD of age was 45.8 ± 10.01. The positive and negative percentages for ESR, CRP, RF, and ACCPs were 59/41, 28/72, 74/26, and 63/37, respectively. Intact PTH level categories were ≤ 65 pg/mL: > 65 pg/mL = 92%:8%.

**Table 1:** Baseline characteristics of the studied sample

*Age (M ± SD) (years)*	45.8 ± 10.01	
***Gender*** *(M / F) n-%*	18/82	18/82
***ESR*** *(+ ve /-ve) n-%*	59/41	59/41
***CRP*** *(+ ve /-ve) n-%*	28/72	28/72
***RF*** *(+ ve /-ve) n-%*	74/26	74/26
***ACCPs*** *(+ ve /-ve) n-%*	63/37	63/37
***Intact PTH level*** *n-%* *(≤ 65 pg/ mL / > 65 pg/ mL)*	92/8	92/8

*+ve = positive, -ve = negative, ESR = erythrocyte sedimentation rate, CRP = C-reactive protein, RF = rheumatoid factor, and ACCPs = anti-cyclic citrullinated peptide antibodies.*

Table (2) shows the FokI genotypes for the cases were FF:Ff:ff = 46%:52%:2%, respectively, and F allele:f allele = 72%:28%. TaqI genotypes for the cases were TT:Tt:tt = 45%:44%:11%, respectively, and T allele:t allele = 67%:33%.

**Table 2:** The distribution of FokI and TaqI genotypes and allele frequencies in the studied sample

VDR genotype n-%					
FokI					
	FF	Ff	ff	F allele	f allele
**n**	46	52	2	144	56
**%**	46	52	2	72	28
**TaqI**					
	**TT**	**Tt**	**tt**	**T allele**	**t allele**
**n**	45	44	11	134	66
**%**	45	44	11	67	33

Table (3) shows that the percentages of positive and negative RF in FokI genotypes were FF:Ff:ff = 45.9%:51.4%:2.7% and 46.2%:53.8%:0%, respectively, with no significant difference among them (P = 1). In TaqI genotypes, the percentages of positive and negative RF were TT:Tt:tt = 40.5%:47.3%:12.2% and 57.7%:34.6%:7.7%, respectively, with no significant difference among them (P = 0.35). These results suggest that there is no association between FokI and TaqI genotypes and RF.

**Table 3:** Positive and negative RF based on the genotype distribution of FokI and TaqI

VDR	Positive		Negative		X2	P
FokI genotype	n	(%)	n	(%)		
**FF**	34	45.9%	12	46.2%		
**Ff**	38	51.4%	14	53.8%	0.39	1
**ff**	2	2.7%	0	0%		
**TaqI genotype**	**n**	**(%)**	**n**	**(%)**		
**TT**	30	40.5%	15	57.7%		
**Tt**	35	47.3%	9	34.6%	2.14	0.35
**tt**	9	12.2%	2	7.7%		

Table (4) shows that the percentages of positive and negative ACCPs in FokI genotypes were FF:Ff:ff = 47.6%:50.8%:1.6% and 43.2%:54.1%:2.7%, respectively, with no significant difference among them (P = 0.9). In TaqI genotypes, the percentages of positive and negative ACCPs were TT:Tt:tt = 44.4%:42.9%:12.7% and 45.9%:45.9%:8.2%, respectively, with no significant difference among them (P = 0.88). These results suggest that there is no association between FokI and TaqI genotypes and ACCPs.

**Table 4:** Positive and negative ACCPs based on the genotype distribution of FokI and TaqI

VDR	Positive		Negative		X2	P
FokI genotype	n	(%)	n	(%)		
**FF**	30	47.6%	16	43.2%		
**Ff**	32	50.8%	20	54.1%	0.6	0.9
**ff**	1	1.6%	1	2.7%		
**TaqI genotype**	**n**	**(%)**	**n**	**(%)**		
**TT**	28	44.4%	17	45.9%		
**Tt**	27	42.9%	17	45.9%	0.47	0.88
**tt**	8	12.7%	3	8.2%		

Table (5) shows that the percentages of ≤ 65 pg/mL to > 65 pg/mL intact PTH level categories in FokI genotypes were FF:Ff:ff = 46.7%:53.3%:0% and 37.5%:37.5%:25%, respectively, with a significant difference among them (P = 0.009). Regarding PTH-level categories, there is a significant difference between ff and FF genotypes and between ff and Ff genotypes, but there is no significant difference between FF and Ff genotypes. The ff genotype had a higher PTH level than the Ff and FF genotypes.

**Table 5:** Intact PTH categories based on the genotype distribution of FokI and TaqI

PTH degree	Fok1							Taq1					
	FF		Ff		ff			TT		Tt		tt	
	n	%	n	%	n	%		n	%	n	%	n	%
**≤ 65 pg/ mL**	43^a^	46.7	49^a^	53.3	0^b^	0		42	45.6	41	44.6	9	9.8
**> 65 pg/ mL**	3^a^	37.5	3^a^	37.5	2^b^	25		3	37.5	3	37.5	2	25
**X2**	10.3						1.94						
**P**	**0.009**						0.36						

*Any value with the letter a differs significantly only with the PTH degree with the letter b.*

In TaqI genotypes, the percentages of ≤ 65 pg/mL to > 65 pg/mL intact PTH level categories were TT:Tt:tt = 45.6%:44.6%:9.8% and 37.5%:37.5%:25%, respectively, with no significant difference among them (P = 0.36).

These results could point out a possible association between the genotype distribution of FokI and the intact PTH degree.

**Fig. 5:**
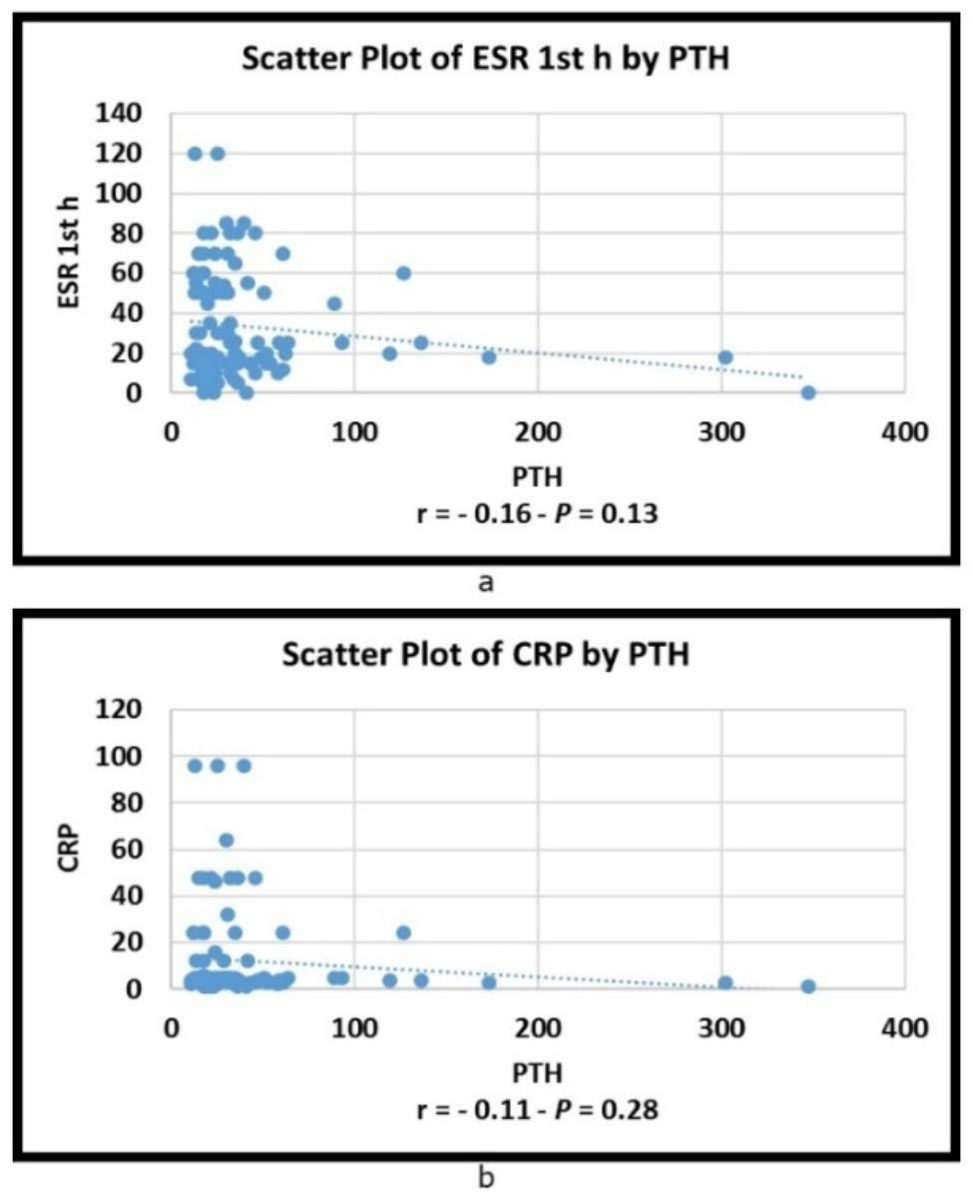
**(a)** Correlation between serum intact PTH level and ESR; **(b)** Correlation between serum intact PTH level and CRP

Figure 5(a) shows that there was no significant correlation between serum intact PTH level and ESR (r = 0.16 and P = 0.13). Figure 5(b) shows that there was no significant correlation between serum intact PTH level and CRP (r = 0.11 and P = 0.28).

**Fig. 6:**
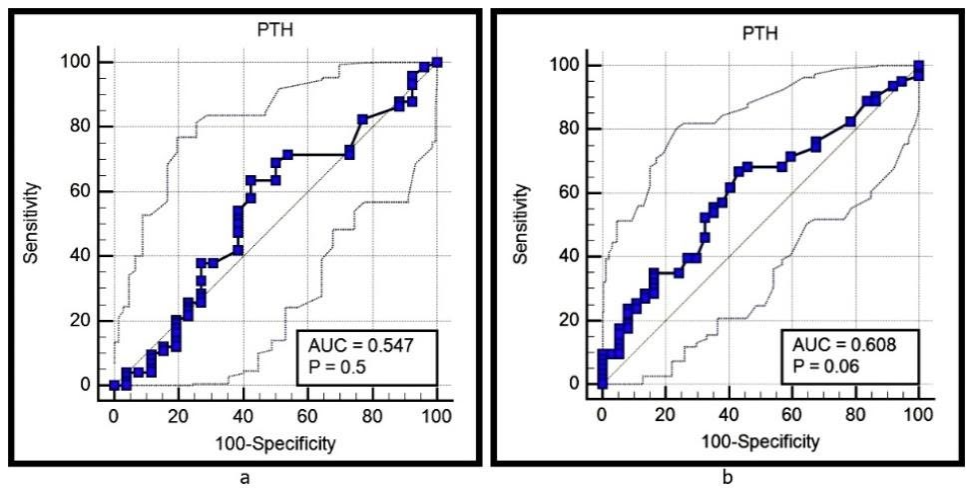
**(a)** Analysis of the ROC curve for intact PTH level as a predictor of positive RF **(b)** Analysis of the ROC curve for intact PTH level as a predictor of positive ACCPs

Figure 6(a) shows that the level of serum intact PTH is not a good predictor for positive RF based on the ROC curve and its area under the curve. The accuracy, sensitivity, specificity, PPV, and NPV were 21.2%, 63.51%, 57.69%, 81%, and 35.7%, respectively, with an AUC of 0.547 (p = 0.5). Figure 6(b) shows that the level of intact PTH is not a good predictor for positive ACCPs based on the ROC curve and its area under the curve. The accuracy, sensitivity, specificity, PPV, and NPV were 23.4%, 66.67%, 56.76%, 72.4%, and 50%, respectively, with an AUC of 0.608 (p = 0.06).

## Discussion

This study was conducted on 100 patients with rheumatoid arthritis. Several results were reached, including that the parathyroid hormone level may be affected by the presence of the disease, as the intact parathyroid hormone increased (> 65 pg/mL) in 8% of the patients.

The combination of primary hyperparathyroidism (PHPT) and rheumatoid arthritis remains controversial; hence, this is not the study’s main focus.

In this study, we found that the F allele was higher in RA cases. To confirm these results, a study examined the relationship between FokI gene polymorphism in rheumatoid arthritis and a healthy control group. The study reported that the FF genotype was significantly higher in rheumatoid patients compared to the control group. Also, the F allele was found to be higher in cases. FF and Ff genotypes led to a higher risk of 7.5 times and 1.5 times, respectively, than having a ff genotype to be a RA patient. The F allele has almost three times the risk of being a RA patient than the f allele [45]. Also, in agreement with the study results about TaqI genotype frequencies in RA, another study revealed nearly similar results, as TaqI genotype frequencies for the RA cases were TT:Tt:tt = 46%:45%:9%, respectively, and T allele:t allele = 68%:32% [46].

The current study revealed that no significant differences among FokI and TaqI genotypes regarding positive and negative RF or ACCPs were found. Another study on RA revealed that there were non-significant differences between different genotypes regarding RF and CRP levels; only the ACCPs level was found to be significantly higher among FF genotypes compared to other genotypes [45]. On the contrary, another study on RA in the Lithuanian population revealed that ACCPs and IL-6 levels were significantly higher in the presence of bb of BsmI genotypes in comparison with Bb and BB (P < 0.001). Moreover, their levels were significantly higher in the presence of FF in FokI genotypes in comparison with Ff and ff genotypes (P < 0.001) [47].

We found a significant difference between percentages of ≤ 65 pg/mL to > 65 pg/mL of serum intact PTH level categories in FokI genotypes (P = 0.009). Agreeing with the results of this study, another study concluded that VDR gene polymorphism influences parathyroid function in chronic renal failure [48]. In RA patients, the correlation between erythrocyte sedimentation rate (ESR) and C-reactive protein (CRP) is 69%. These measures are nearly equal in predicting swollen joint count and may be correlated with clinical disease activity [49].

In the present study, there was no significant correlation between serum intact parathyroid hormone levels and ESR or CRP. Agreeing with these results, a study revealed that normal levels of basal IL-6 and CRP in PHPT patients when compared to healthy controls have been reported, which indicates a weak or absent role of PTH in controlling the level of these biomarkers [50, 51].

On the contrary, a study concluded that the mean serum concentrations of IL-6, CRP, and ESR had increased one year after surgical cure of the parathyroid disease (p < 0.001, p < 0.01, and p < 0.001, respectively) [51]. It is unclear how serum PTH and inflammatory markers like IL-6 and CRP relate to each other. Uncertainty exists regarding the connection between PTH and inflammation [52]. Some studies showed a paradoxical rise in CRP levels following parathyroid surgery [51, 53, 54]. PTH levels were significantly lower in patients with CRP > 60 mg/liter compared to patients with CRP < 20 mg/liter [55]. PTH level is negatively correlated to serum levels of ESR and CRP (r = -0.609 and − 0.711, respectively), with a significant value (p < 0.01) for both results [56]. A study found a positive association between PTH and CRP after adjustment for multiple potential confounders (all 𝑃 < 0.001) [52]. Asymptomatic PHPT patients had considerably higher levels of hs-CRP and IL-6 than did the control [57].

Previous studies of PHPT have shown normal [50, 58] or increased levels of basal IL-6 and/or CRP compared to healthy controls [59–62]. Studies involving both pre-operative and post-operative measurements of IL-6 and/or CRP in patients treated for PHPT have reported conflicting results showing decreased [59], increased [54, 60], or unchanged [61, 63] levels.

## Limitations

Ethnic differences have an effect on VDR genes and RA etiopathogenesis, so this study should be conducted in many countries. The presence of a healthy control group would greatly improve the result analysis.

## Conclusion

This study demonstrated that there is an increase in serum intact PTH level in 8% of rheumatoid arthritis patients, which indicates an imbalance in the level of the hormone as a result or cause of RA. FokI and TaqI genotypes had no effect on biochemical markers (RF and ACCPs) in rheumatoid arthritis patients. FokI but not TaqI genotypes had an effect on serum intact PTH level in rheumatoid arthritis patients. The potential clinical significance of the results obtained is that there is a possible genetic role in determining PTH level in RA. Serum intact PTH level in rheumatoid arthritis patients did not correlate to ESR, CRP, RF, or ACCPs in rheumatoid arthritis patients, so serum intact PTH level did not predict susceptibility to RA or disease severity according to the previous biomarkers.

## Data Availability

The datasets generated and/or analyzed in this study are available from the corresponding author on reasonable request.
